# Sarcopenia: What Is the Origin of This Aging-Induced Disorder?

**DOI:** 10.3389/fgene.2021.688526

**Published:** 2021-07-02

**Authors:** Thomas Gustafsson, Brun Ulfhake

**Affiliations:** Division of Clinical Physiology, Department of Laboratory Medicine, Karolinska Institutet, Stockholm, Sweden

**Keywords:** muscle weakness, muscle mass, aging, demography, heritability, phenotype, genotype

## Abstract

We here review the loss of muscle function and mass (sarcopenia) in the framework of human healthspan and lifespan, and mechanisms involved in aging. The rapidly changing composition of the human population will impact the incidence and the prevalence of aging-induced disorders such as sarcopenia and, henceforth, efforts to narrow the gap between healthspan and lifespan should have top priority. There are substantial knowledge gaps in our understanding of aging. Heritability is estimated to account for only 25% of lifespan length. However, as we push the expected lifespan at birth toward those that we consider long-lived, the genetics of aging may become increasingly important. Linkage studies of genetic polymorphisms to both the susceptibility and aggressiveness of sarcopenia are still missing. Such information is needed to shed light on the large variability in clinical outcomes between individuals and why some respond to interventions while others do not. We here make a case for the concept that sarcopenia has a neurogenic origin and that in manifest sarcopenia, nerve and myofibers enter into a vicious cycle that will escalate the disease progression. We point to gaps in knowledge, for example the crosstalk between the motor axon, terminal Schwann cell, and myofiber in the denervation processes that leads to a loss of motor units and muscle weakness. Further, we argue that the operational definition of sarcopenia should be complemented with dynamic metrics that, along with validated biomarkers, may facilitate early preclinical diagnosis of individuals vulnerable to develop advanced sarcopenia. We argue that preventive measures are likely to be more effective to counter act aging-induced disorders than efforts to treat manifest clinical conditions. To achieve compliance with a prescription of preventive measures that may be life-long, we need to identify reliable predictors to design rational and convincing interventions.

## Introduction

*Aging* is commonly used to name the progressive decay of organism’s function in later life. Aging has an indistinct onset and a highly variable inter-individual progression. Today as well as in all recovered marks of human culture the inevitable end of life has provided a strong incentive to find measures to postpone it. While substantial progress has been made in extending the average expected lifespan, rejuvenation (or preserving youthfulness) still remains a concept more than a reality. The extension of lifespan among those we consider long-lived is not very impressive compared with the gain for those that previously died at a younger age. The latter accomplishment came by improving living conditions and by preventing as well as combating major health threats until modern times, poor nutrition, and infectious diseases. Throughout the post-industrial nations and now also in developing countries, this has led to a dramatic change in the population composition by age (demography). The inflation of the aged over the younger cohort and the exponential growth of care expenditures, lead to an economic and social stress on society that needs to be addressed. Focus has therefore shifted from lifespan to healthspan, to extend the years that older people can continue to be independent, participate and contribute to society rather than experience life quality deterioration and consume resources because of poor health. To meet these challenges, the WHO recently classified aging-induced disorders as diseases (World Health Organization (WHO)^2019^, ^2020^), an action intended to pave the way for the development of rational interventions to impede or even halt the aging-induced decline of health (The Lancet Diabetes Endocrinology (LDE), 2018).

In this *future perspective* we will touch upon the recent major demographic changes and biological processes considered to be involved in aging, and then devote the main part to sarcopenia, i.e., the dissipation of muscle function and mass, a conspicuous trait of the aged human phenotype. We chose sarcopenia because it is a facultative decay occurring also in healthy aging individuals yet it is accelerated by comorbidity, hospitalization, and a sedentary lifestyle. According to World Health Organization (WHO) (2000), sarcopenia is one of the major causes of loss of independence and a very significant risk factor for developing other morbidities at older age.

## Lifespan and Healthspan

**FIGURE 1:**
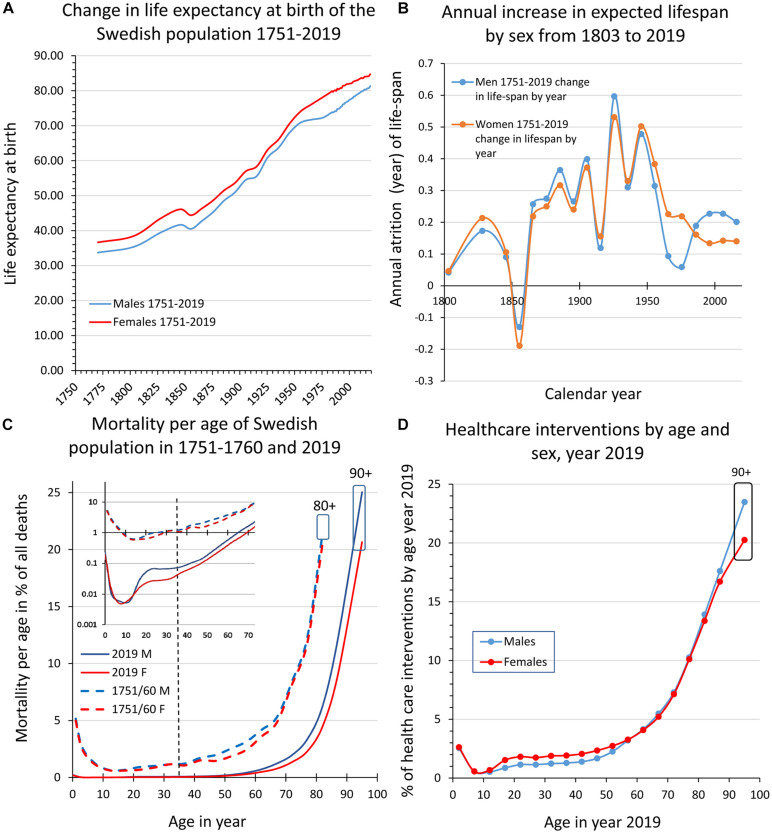
Swedish population statistics 1751–2019. **(A)** Shows the increase in expected lifespan at birth for women and men. **(B)** Diagram illustrating the increase (averaged across 10 years bins) of expected lifespan at birth by year and sex 1803–2019. **(C)** Shows the mortality (%) by age and sex in 1751–1760 and in 2019, respectively. Inset in **(C)** is part of the main diagram but with a logarithmic scale on the ordinate. The curves illustrate that the major impacts on lifespan for women and men are the decreased mortality in early life and at ages 50–80 years, while mortality today is still high at >80 years and corresponds to the leveling-off of lifespan increase evident in more recent data (c.f., **A,B**). **(D)** Shows health care interventions by age and sex in 2019 where the decreased mortality in early and late ife **(C)** corresponds to higher levels of health care consumption. (**A–D** are data replotted from Statistics Sweden (1999) and http://www.statistikdatabasen.scb.se/pxweb/en/ssd/).

Over the past one and a half centuries, the average human life span has almost doubled in high-income countries (HICs) and middle-and-low-income countries (MILOICs) are following. According to the Gerontology research group^1^, a low boundary estimate of the number of people alive today at older age than 109 years is close to 500. They are all born early in the 20th century and since then the world population has increased from 1.6 to 7.8 billion (United Nations (UN), 2019). Due to this enormous increase in human phenotypes (molded out of the genotypes), it is believed that the number of supercentenarians will grow rapidly and that the apparent ceiling of human longevity around 120 years will be pushed forward. However, this raises issues with the interpretation of the lifespan extension during the 19th–20th centuries and the scarcity of reliable more distant historic data as well as to what extent longevity is heritable (Oeppen and Vaupel, 2002; Crimmins, 2015; Dong et al., 2016; Kontis et al., 2017; Ben-Haim et al., 2018; Kaplanis et al., 2018). As Sweden has one of the best backlogging population statistics, we will use this here as a framework to discuss changes to lifespan and mortality across the past centuries and, in addition, how health care intervention is consumed by age and sex in the year 2019 (Figure 1).

Since 1751, life expectancy at birth has more than doubled in Sweden (Figure 1A). The increase was particularly fast in the 20th century (Figure 1B) but as we approach and surpass 80 years in lifespan expectancy at birth (Figure 1A) and in agreement with several other HICs, lifespan extension is now slowing down (Figures 1A,B; Dong et al., 2016). By comparing mortality by age and sex we can see that the extended lifespan is due to the curbing of early life mortality (see inset in Figure 1C; <5 years; 3–5% 1751–1760 down to 0.01–0.32% in 2019) meaning that today with a few exceptions all those born alive will reach puberty. At menarche/puberty and throughout a major portion of the reproductive period (here defined as the period between 15 and 49 years), mortality remains low and remarkably stable at 0.6–1.2% in 1751–1760 and 0.2–0.6% in 2019 until age 35–39. This “reproductive mortality plateau” present in the Swedish population statistics both from 1751–1760 and 2019, is in no way exclusive to Sweden (Perls et al., 2002; Crimmins, 2015) and suggests that the human population has been selected from those that had good health through a sufficiently long period to reproduce and nurture a surplus of children also under more challenging and impoverished conditions^2^. The second major change is the shift from ∼50 to ∼70 years of age as the breakpoint when mortality start to increase rapidly (Figure 1A); at above 75–80 years of age the mortality is high still today (*idem*). When comparing the changes in mortality between 1751–1760 and 2019 (Figure 1C) with health care interventions by age and sex in 2019 (Figure 1D), we can conclude that the much-improved survival in early life and at older age both are reflected in a comparatively larger consumption of health care interventions of these age groups. This indicates that we have been more successful in curbing mortality than to decrease morbidity (here defined as an instance of intervention with a treatment diagnosis) over the past centuries and consequently that aging is a key co-factor for the concurrent and future health provisions by society (Ben-Haim et al., 2018; Partridge et al., 2018). Lifespan extension and the parallel change to the fertility rate (Supplementary Figure 1A) have altered the demographic profile toward older ages (Supplementary Figure 1B), forcing society to recognize aging as a major factor for dependence and morbidity. In order to facilitate the development of effective preventive medicine that can postpone morbidity and increase healthspan, we need reliable predictive biomarkers of morbidity that also consider the impact of aging (Beekman et al., 2010; Deelen et al., 2013).

## Biology of Aging

**FIGURE 2:**
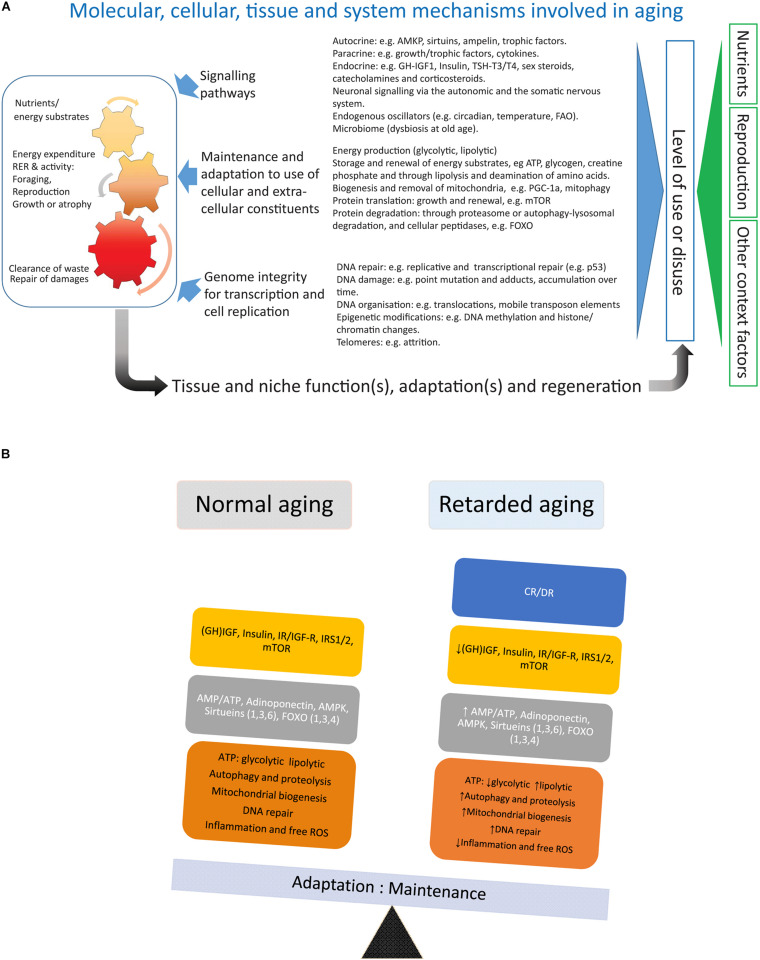
**(A)** Depicts factors involved in the aging of an organism. **(B)** Summarizes the main effects of calorie/dietary restriction.

A very simplistic description is that aging is the accumulation of non-degraded waste (e.g., myelin bodies, lipofuscin, and amyloid) and unrepaired damage to cellular/extracellular constituents with a run-down of cellular energy production, and the gradual exhaustion of the replicative capacity forcing cells into state of non-responsiveness/senescence (Figure 2). This gradual decay has a variable pace both between tissues within individuals as well as between individuals and lead to the disintegration and finally death of the organism. Why aging occurs is a much debated issue (Kirkwood and Melov, 2011; Azzu and Valencak, 2017) but humans in HICs currently live more than twice the time it takes to reproduce and nurture a growing human population and the older ages beyond the reproductive era make up an increasingly large part of the lifespan strongly associated with morbidity and decay of function. While any benefit for the human species of growing old remains to be evidenced, closing the gap between health- and lifespan is not an issue of opposing views. Studies devoted to shed light on the heritability of growing old indicate that only a modest 25% of the variability in lifespan can be accounted for by heritage (Wijsman et al., 2011; Kaplanis et al., 2018; Partridge et al., 2018). However, as we push the expected lifespan toward the ages of those that are most long-lived (100+) and who did not experience the same increase in lifespan over the past centuries, the genetic constraint of human lifespan may become increasingly important (Barzilai et al., 2010; Beekman et al., 2010; Kenyon, 2010; Rajpathak et al., 2011). This assumption is supported by the fact that most long-lived humans appear to be protected against some of the hazards conferring morbidity and death among more short-lived humans (Beekman et al., 2010; Rajpathak et al., 2011). Aging is a complex polygenic trait and already the non-exhaustive listing of 24 genes with a strong association with longevity in humans (Partridge et al., 2018) is extensive enough to generate a very large number of different genotypes and, when coupled with environmental impact, an even larger set of phenotypes. To close some of the gap between health- and lifespan at older ages, a deeper understanding of the genetics of aging is needed (Atwood et al., 2003; Kirkwood et al., 2011).

Calorie/dietary restriction (CR/DR) is the most robust and general (across mammalian and non-mammalian species) intervention known to retard aging and is therefore a useful tool to dissect the processes underlying aging and aging-induced traits like sarcopenia (an extended review of CR with references is available in the Supplementary Material). Laboratory work in the early 20th century (for references see Speakman and Mitchell, 2011) showed that reducing calorie or dietary (CR/DR restriction) intake by 10%-45% increased lifespan by up to 50% in many invertebrate and vertebrate species, implicating that this intervention intercepted with highly conserved mechanisms of systems biology involved in the aging of organisms. In mammals, CR reduces the incidence of common morbidities such as cardiovascular diseases and cancer (main causes of death at older ages in HICs) and, thus, seems to extend healthspan to a similar extent as lifespan. In mammals like small rodents CR will retard body growth and, if initiated in adulthood, lower body weight, an adaptation which will affect both lean body mass and, in particular, adipose tissue (Duffy et al., 1997; Cameron et al., 2011). Rodents are endothermic and respond to CR with a lowering of body temperature and torpor (*idem*), accompanied by alterations in the endocrine system with decreased levels of TSH, T4, and T3 (Araujo et al., 2009) (HPT axis), growth hormone and IGF-1, and lower levels of blood glucose and insulin as well as increased insulin sensitivity (Cameron et al., 2011; Azzu and Valencak, 2017). Metabolically, there is a shift toward β-oxidation of fatty acids (Bruss et al., 2010). This metabolic shift has also been argued to be the main reason for the lower levels of oxidative (ROS) stress in subjects under CR (Speakman and Mitchell, 2011). The increase in metabolic activity of white adipose tissue (WAT) under CR is accompanied by an upregulation of key lipid enzymes and a browning of the white fat (Bruss et al., 2010; Fabbiano et al., 2016). The decrease in amount of WAT under CR also lowers levels of leptin and increases levels of adiponectin (Hardie, 2011). The decreased production of ATP (higher AMP:ATP ratio) and increased adiponectin signaling will both induce AMP-activated protein kinase (AMPK) (Hardie, 2011), which will depress the anabolic drive by inhibition of mTOR (*idem*). mTOR (mammalian/mechanistic target of rapamycin) is a key protein of the TORC complex (TORC1/2) regulating cell growth by promoting protein translation and inhibiting the FOXO family of transcription factors (*idem*). In addition to inhibiting mTOR through inducing AMPK and decreasing insulin/IGF-1 signaling, CR induces also another family of proteins, the sirtuins (Sirt 1-6) (Guarente, 2011). The sirtuins [nicotinamide adenine dinucleotide (NAD)-dependent protein deacetylases] are metabolic sensors that stimulate mitochondrial biogenesis, lower oxidative stress, inhibit mTOR and induce both FOXO and autophagy-lysosomal activity, reduce oxidative stress and DNA damage, and increase p53 activity (*idem*). Thus both actions by the sirtuins and the AMPK-driven inhibition of mTOR will activate the FOXO family of transcription factors, paving the way for increased regulated proteolysis through the ubiquitin-proteasome system (UPS) and increased degradation of proteins, molecular complexes, and organelles through the autophagy-lysosomal pathway conferring a more rapid turnover of cellular constituents (Martins et al., 2016; Maiese, 2021). FOXO proteins also upregulate cellular oxidative defense and have an inhibiting function on proteins involved in cell cycling (*idem*). Combined, the CR-mediated decrease in oxidative stress, increased biogenesis of mitochondria, and accelerated turnover of cellular constituents may slow the wear and tear that generates the cumulative build-up of unrepaired damage during aging. Trials with chemical compounds that show the promising extension of lifespan in model organisms are all partial mimetics of CR and targets mTOR (rapamycin, Miller et al., 2014), AMPK (metformin, Weiss et al., 2018), or indirectly both sirtuins and AMPK by inhibiting mitochondrial function (resveratrol, Price et al., 2012).

Trials with CR on long-lived species such as primates (Colman et al., 2009; McKiernan et al., 2011) and humans (Most et al., 2017) remain limited, for obvious reasons. However, those to date show promising outcomes, suggesting that CR mediates its effects through the same evolutionary conserved mechanisms as described above for small rodents. Furthermore, several of the gene variants associated with human longevity (Partridge et al., 2018) are genes implicated in the beneficial outcome of CR, such as FOXO(3A), the growth hormone-IGF-1 signal transduction pathway, and proteins involved in cell cycling (Guevara-Aguirre et al., 2018).

## Sarcopenia

### What Is Sarcopenia?

**FIGURE 3:**
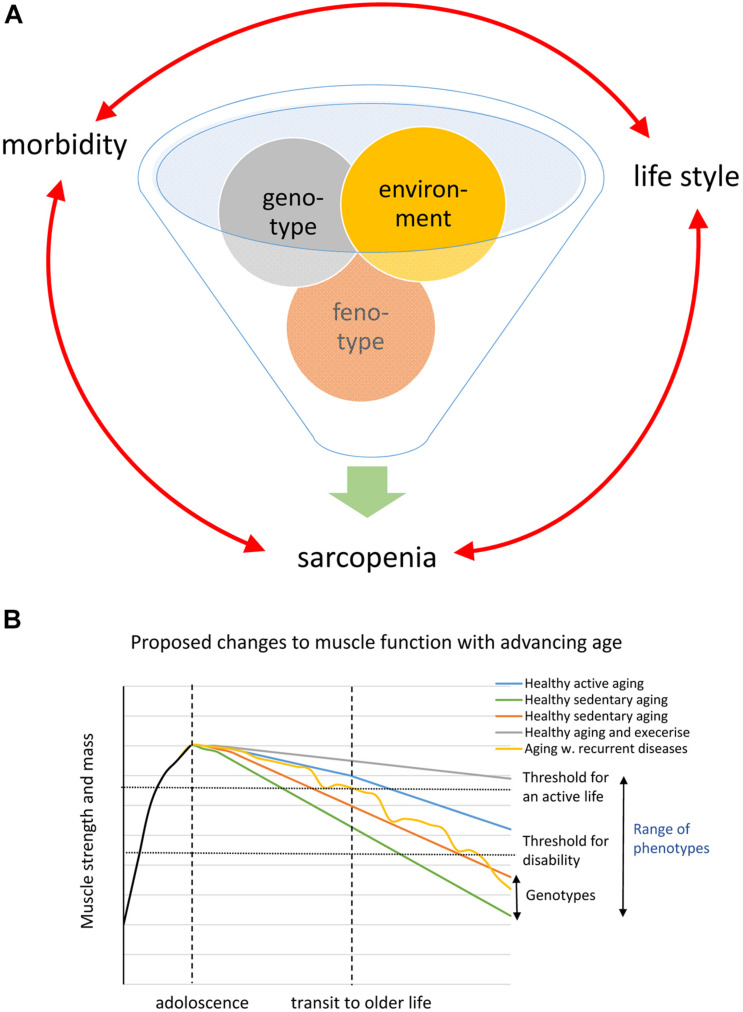
**(A)** Schematic of the inferred interaction genotype and environment generating the phenotype. **(B)** Shows a schematic drawing based on the WHO diagram illustrating different possible trajectories caused by genotype and phenotype in the loss of muscle strength and mass during aging.

**FIGURE 4:**
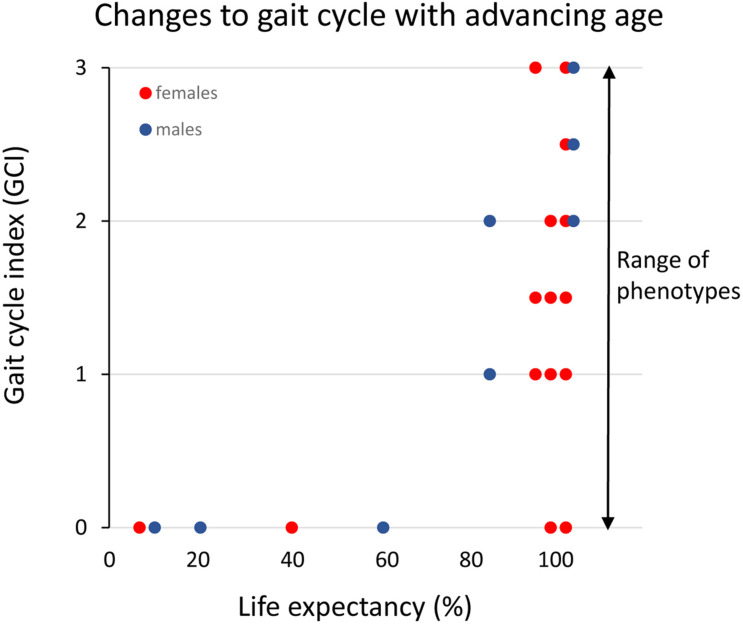
Clinical assessment of gait cycle aberrations in small rodents across the median lifespan in percent (data replotted from Altun et al., 2007 and unpublished data). The different gait cycles phases and the coordination between limbs are preserved (index = 0) until the final 20% of the life-span when these functions progressively deteriorate. However, note that there is a large variance among individual rats in the progression of this aging-induced impairment and that some very old rats still have an intact gait cycle.

A conspicuous trait of the aged phenotype is the reduced function and mass of skeletal muscles, which became a clinical entity (Evans, 1995) coined sarcopenia in 1989 because of the dramatic increase in incidence and prevalence brought about by the demographic changes of the 20th century (see above). Sarcopenia was recognized by the WHO (World Health Organization (WHO), 2000) in 2000 as a major threat to independence and as a risk factor for multiple morbidities associated with advanced age (Figure 3A) and targeted because it may be modifiable by rational lifestyle interventions (Roubenoff, 2000; Figure 3B). As mentioned above, sarcopenia is now a disease entity with an ICD-10-CM code (M62.84). Later, sarcopenia became a corner stone of the clinical concept “frailty” introduced to define more broadly the vulnerability of old individuals (Roubenoff, 2000). As a clinical entity, sarcopenia needs a rigorous operational definition to facilitate the coherent design of epidemiological, clinical, and experimental studies as well as assessment of interventions. However, this issue was not addressed in full until about a decade ago. The two most well-known sets of operational criteria for sarcopenia today are the EWGSOP2 (Cruz-Jentoft et al., 2019) and the FNIH (Studenski et al., 2014). Although these two definitions differ to some extent, they both define primary sarcopenia (in aging of healthy subjects) as the graded loss of skeletal muscle mass, decrease in muscle strength (most commonly static handgrip strength), and performance (usually gait speed with a set distance of 400 m) (*idem*). The latter is highly relevant because of the claimed link between sarcopenia and high risk of (serious) fall accidents among the elderly. While gait is a locomotor behavior relying on timed integration of sensory input and motor command is a clinically relevant assessment of muscle coordination and function; critique of the concurrent definitions of sarcopenia [as will be discussed below] has been raised in particular toward the emphasis on muscle mass but also the use of static muscle strength rather than dynamic muscle force measures (McGregor et al., 2014; Tieland et al., 2018). Furthermore, gait translates well to corresponding read outs in experimental animal models of sarcopenia (Altun et al., 2007; Fahlstrom et al., 2012; Bair et al., 2019; Figure 4). In the literature, the incidence, prevalence, and grading of severity as well as the clinical progression of the condition varies substantially, but studies are reasonably coherent on the evidence that primary sarcopenia becomes clinically assessable during the 5th–7th decades of life with a prevalence of >5%, with an annual progression of 0.5–2% per year (using above mass and strength criteria), and an accelerated progression beyond 70 years with a prevalence of >10%. Confounding factors, apart from differences in operational definitions and the relevance of the metrics used to define and grade sarcopenia, are problems associated with study design, either cross-sectional or longitudinal (Reid et al., 2014; Skoglund et al., 2020), and whether the studied populations are representative of the general population (Mitchell et al., 2012).

#### Concepts of Sarcopenia

There is still an ongoing debate about whether sarcopenia has a neurogenic or myogenic origin (Gutman and Hanzlikova, 1972). At more advanced stages of the disease, a multitude of alterations are present in the peripheral innervation as well as in the target tissue, which makes it quite challenging to determine cause and effect.

#### Neurogenic Mechanisms of Sarcopenia

##### Loss and re-organization of motor units during aging and impact on muscle mass, force, and power

A century ago, Liddell and Sherrington (1925) established that the grading of muscle force is by the recruitment of sets of myofibers, each innervated by one α-motoneuron (MN)—collectively referred to as a motor unit (MU). Subsequently, MUs were categorized according to differences in mechanical properties. At one end of the spectrum, are the slow twitch fatigue-resistant MUs (type S) with type I myofibers and at the other end, fast twitching rapidly fatigable MUs with type II (IIb; type FF) myofibers (Burke, 1999). Intermediate types include the transitional forms that are fast twitching but resistant to fatigue with type II (IIa and IIx; types FR and FI) myofibers (*idem*). Small muscles comprise usually proportionally fewer (<100) MUs than a large muscle (up 700–800 MU) (Feinstein et al., 1955; Gath and Stålberg, 1981). The MU populations hold MUs with a highly variable number of myofibers (from <10 up to >1000, depending on muscle type). S-type MU may have 30–120 fibers while fast fatigable (FF) MUs have several hundred up to more than one thousand fibers (Feinstein et al., 1955). Regardless of MU type, the fibers of an individual MU are all of the same type and for mechanical reasons dispersed across a larger territory of the muscle cross-section (Edström and Kugelberg, 1968; Burke, 1999; Larsson et al., 2019). In the seminal paper by Buller et al. (1960), evidence was provided that the innervating MNs exert a powerful influence on the phenotype of myofibers (being slow or fast twitch). This organizational design where a skeletal muscle contains a pool of MUs with different mechanical properties including size, provides for a fine tuning of muscle force output adequate and economical for each locomotor task. As recorded from live animals (Hennig and Lomo, 1985), everyday motor tasks including walking and stairclimbing with long duty cycles involve recruitment of mainly S type MU, while moving with higher speed and running expand the recruitment more toward fast fatigue-resistant (FR) MUs and only in brief ballistic movements also the fast fatigable MUs are recruited in mixed muscles (Hennig and Lomo, 1985; Burke, 1999). Thus, in contrast to the S type MU, duty cycles are brief and quite infrequent among fast and fatigable MUs (*idem*).

Recordings from aged animals and humans alike, consistently show that the number of MUs decreases with advancing age but importantly, also that MUs are re-modeled. The latter is due to a re-innervation of denervated fibers similar to the process that is invoked by accidental or experimental severance of the muscle innervation and referred to as collateral re-innervation (McNeil et al., 2005; Power et al., 2016). Based on changes in peripheral nerve conduction velocity, latency, amplitude, and time course of the myofibers response recorded by EMG and muscle histology, available data show that aging-induced atrophy preferentially but not exclusively affects type II myofibers (Larsson, 1978; Lexell et al., 1983). Type II fibers display additional signs of denervation by re-expressing the Na_*v*_1.5 sodium channel, the CHRN-γ subunit, and embryonic and neonatal isoforms of myosin. If such a fiber is re-innervated by an S type MN the fiber will co-expresses slow MyHC (hybrid fibers) and is spatially clustered to nearby slow fibers (fiber type grouping) enlarging S type MUs at the expense of functional F type MUs (Rowan et al., 2012; Piasecki et al., 2018). Slow muscles (e.g., m. soleus) and type I myofibers are, however, not spared from sarcopenia (Purves-Smith et al., 2012).

**FIGURE 5:**
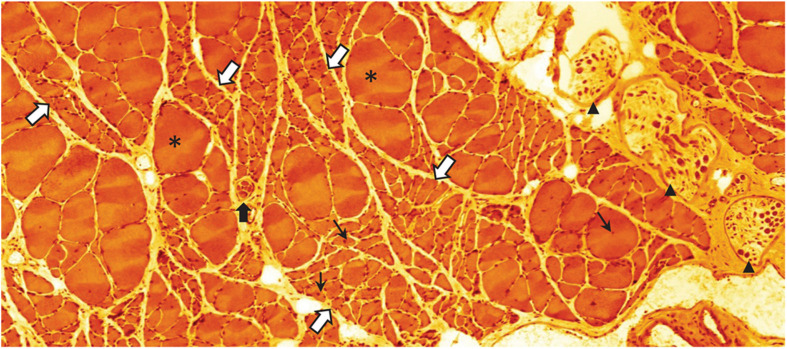
Eosin-hematoxylin stained sections through m. soleus of a 30-month-old rat (average life expectancy for this strain) suffering from severe sarcopenia. Bundles of normal appearing and occasional extremely hypertrophic (asterisks) myofibers are intermingled with bundles of severely atrophied myofibers (white arrows with blue border). Normal, atrophic, and hypertrophic myofibers also have nuclei with a central location (small black arrows) indicating that these nuclei have recently been recruited to the myofiber from the satellite cell pool. This histological appearance is very similar to what can be seen following traumatic or accidental injury to the muscle nerve. Black arrow heads point to intramuscular branch profiles of the muscle nerve. Notice the low density of large axon profiles. The black arrow with white border indicates a muscle-spindle, a sensory organ that records the tension of the muscle (unpublished image by BU).

As the sarcopenia process progresses, the collateral re-innervation mechanism will be exhausted and the loss of force and mass will become clinically overt (McNeil et al., 2005; Kawabuchi et al., 2011; Piasecki et al., 2018). Thus, clusters of severely atrophic—probably completely dysfunctional—fibers are a frequent observation in advanced stages of sarcopenia (Altun et al., 2012; Figure 5). Combined, the alterations of the MU pool composition and size will affect several qualities of motor behavior that should be possible to assess in the aging subject: a decline in power and force (preferential loss of fast MUs), decrease of maximum speed of contraction (increasing predominance of S MUs), and because of the increasing size of S MUs (through collateral re-innervation), fewer fine-tuned adjustments of locomotor tasks (McNeil et al., 2005; Piasecki et al., 2018). A distinctive difference between the denervation taking place in sarcopenia during normal aging and that occurring in motor neuron diseases like ALS, is the absence of fibrillations in sarcopenic muscles; suggesting that the denervation and re-innervation processes may be different (Wohlfart, 1957).

##### Changes in the peripheral innervation during aging and drop out or dying back of MNs

Skeletal muscles receive sensory innervation and both somatic and autonomic efferent innervation. Skeletal muscle function relies on a preserved innervation but degenerative changes in the peripheral nervous system are wide-spread and progressive during aging (reviewed in Cowen et al., 2005). These include loss of innervation, loss of peripheral sensory receptor organs, axonal atrophy and dystrophy, and aberrations to the myelin. In many respects, these changes are similar to those observed in the central nervous system albeit the environmental distinctions between these two compartments (Burek et al., 1976; Ramirez-Leon et al., 1999). Myelin de-and dys-myelination during aging seem to cause a tissue loading of non-disposed myelin breakdown products, which drives inflammation through interferon-γ/TNFα signaling (Kullberg et al., 1998; Edstrom et al., 2004; Safaiyan et al., 2016). Myelin aberrations may also impact axon impulse conduction velocity and the conduction safety factor making impulse traffic less predictable and well-timed (McNeil et al., 2005). Due to the structural alterations of the myelin sheaths and the axonal atrophy/dystrophy, it is challenging to assess losses of specific subsets of axons in the peripheral muscle nerve during aging. In addition, there is ample evidence that the sensory feedback is likewise affected (reviewed in Cowen et al., 2005) and studies on aging-associated changes in peripheral sensory/motor nerves and muscle force seem to be in agreement (Strotmeyer et al., 2009; Ward et al., 2015).

Due to all the technical difficulties associated with unbiased counting of MNs in the spinal cord (e.g., in humans, cadaver examination using only anatomy-based identifiers), the estimation of MN numbers lost during aging varies considerably between studies (<15% up to 30–50%) but it is likely on the modest side of the range and in-line with assessment of loss of MN axons in peripheral nerves (Kawamura et al., 1977; Tomlinson and Irving, 1977). Results obtained by using a retrograde transported tracer taken up by motor axons in aged small rodents with sarcopenia suggest that up to 30% of the MNs are lost, or have impaired axonal transport and, further, that this decrease is more accentuated in the lower than the upper limb (Hashizume and Kanda, 1995). This is consistent with observations that loss of strength and power is more conspicuous in muscles of the lower compared to the upper limbs. Evidence that MNs lose contact partially or in full, with the target myofibers and, in parallel, other MNs that engage in axonal sprouting to re-innervate the vacated myofibers have support in the phenotypic changes observed in the cell bodies of spinal cord MNs of small rodents during aging (Johnson et al., 1995).

The stronger susceptibility to aging of fast twitch over slow twitch MUs may relate to the difference in number of target myofibers (up to 20x higher). Thus, the extensive and elaborate terminal axon arborization of fast MUs may generate a correspondingly larger burden for maintenance. Furthermore, their infrequent use in everyday locomotor activities may add to this. Alluding to the burden of maintenance due to size and complexity, available evidence suggests that distal projecting MNs suffer more from aging than those that project proximally. It should be recognized that the long peripheral trajectory of an MN to distal limb MUs may well exceed 1 m, making it more vulnerable to mechanical wear and tear along its path (Thomas et al., 1980).

##### The neuromuscular junction (NMJ)

The NMJ is a synapse, perhaps one of the most well-studied (Katz and Miledi, 1965), and the components of the NMJ, its development, and normal function have been discussed in detail elsewhere (for recent reviews see Tintignac et al., 2015; Willadt et al., 2018). In short, the action potential (AP) of the motor axon is transmitted through the release of acetylcholine (ACh) quanta acting on post synaptic nicotinic ACh receptors (CHRNs) are pentamers assembled from four different types of subunits (α, β, δ, and ϵ), to generate an excitatory end plate potential (EPP) which will initiate the contraction process of the myofiber. The signal is disrupted by enzymatic cleavage of the transmitter and re-uptake by a transporter to the presynaptic ending. In between APs, there is a leakage of ACh quanta generating miniature end plate potentials (mEPP) (*idem*). The importance of the mEPPs in between APs was not understood until it was shown that these mEPPs as well as leakage of ACh quanta from disconnected axons serve the purpose of maintaining the integrity of the end plate and facilitate re-innervation of vacated myofibers (Vyskocil et al., 1995). In addition to ACh, motor axons secrete agrin and α-calcitonin-gene-related peptide (CGRP). Agrin instructs the myofiber by interaction with the muscle-derived LRP4/MuSK complex to assemble and concentrate CHRNs into the folded subsynaptic sarcolemma and to build the complex sub-sarcolemmal molecular structure of the end plate. The role of CGRP is still unclear but has been proposed to maintain the end plate in situations of denervation (Machado et al., 2019) and to increase ACh quantal size (Gaydukov et al., 2016); furthermore, CGRP seems to be preferentially expressed by the less frequently recruited MNs of fast MUs (Piehl et al., 1993) and is massively expressed by MNs disconnected from their target or engaged in collateral re-innervation (Johnson and Duberley, 1998). Although convincing evidence is missing, CGRP may have a function similar to the spontaneous quantal release of ACh, i.e., to preserve the end plate integrity during periods of disuse or denervation.

Conversely, the target muscle produces trophic factors (e.g., CNTF, GDNF, NGF, BDNF, and more, reviewed in Kawabuchi et al., 2011; Mrówczyński, 2019; Stanga et al., 2020) that are taken up and retrogradely transported to the MN cell body. These neurotrophins are non-redundant for MN survival during development (Hollyday and Hamburger, 1976) but are continuously expressed throughout life and, as suggested by experimental data, may then serve different functions such as potentiation of transmission and frequency of mEPPs (Gaydukov et al., 2019) (BDNF), maintenance and re-innervation (Stanga et al., 2020) (GDNF), and MN maintenance (BDNF, GDNF; *idem*). Assessment in aged MNs (and sensory neurons) of the expression of target-derived neurotrophic factor receptors of the NGF family and the expression level of these neurotrophic factors in the target tissue points toward decreased trophic support in old age (Edström et al., 2007). In contrast, GDNF, which has also been implicated in maintenance and regeneration of the NMJ, is increased in aged muscles, and the cognate tyrosine kinase receptor c-ret is upregulated in the innervating MNs (idem). Myofiber and/or Schwann cell-derived GDNF also interacts with the GRF-1α receptor and NCAM in the myofiber, and NCAM has been proposed as an interaction partner in collateral re-innervation processes (Kawabuchi et al., 2011). Another player at the NMJ is the population of terminal Schwann cells (TSCs). Ablation of this cell population impairs innervation, collateral re-innervation, and removal of axon endings (Nishimune and Shigemoto, 2018). TSCs provide a leading edge that aid axon sprouts to reach the site of a vacated end plate and also appear to have a protective function on the NMJ (Love and Thompson, 1999; Kawabuchi et al., 2001; Kang et al., 2019). Some data indicate that aging impairs these functions (Love et al., 2003) and that the integrity of the NMJ depends on activity (Love and Thompson, 1999; Soendenbroe et al., 2020). When engaged in collateral re-innervation, both the growing sprout and the TSC express the growth-associated protein 43 (GAP43) by which they can be identified. MNs in the aged rodent spinal cord contain highly increased levels of both CGRP and GAP43, suggesting that a large fraction of the MNs are involved in the denervation and re-innervation processes of the target muscles (Johnson et al., 1995). Moreover, the concurrent regressive impact by aging on autonomic efferent innervation (Cowen et al., 2005) may also adversely impact NMJ transmission since catecholamines released by autonomic nerve endings via parasynaptic action can modulate both the EPP and the mEPP at the NMJ and is used as an adjuvant therapy in conditions of myasthenia gravis (Vanhaesebrouck et al., 2019).

With advancing age the NMJ displays multiple molecular and structural alterations (see above) including the removal of pre-terminal axons and disintegration of end plates (Kawabuchi et al., 2001; Rowan et al., 2012). There are conflicting opinions about what instigates the denervation process; is it due to impairment of the motor axon or the target myofiber? However, if we consider that many myofibers are successfully re-innervated by sprouting axons until they clinically manifest as sarcopenia, the natural history argues strongly against a primary myofiber origin. In sarcopenia, the motor axon ending is removed from the NMJ by TSC, however, the signal triggering this event remains unknown (Yin et al., 2004). Subsequently, the vacated end plates may become re-innervated by collateral sprouting from nearby intact axons through guidance by the TSC. While we know that this happens, we do not understand the non-redundant signals in-between the denervated fiber, the TSC, and the axon sprout. Unraveling the crosstalk that drives these events need to be prioritized because they all represent potential targets for intervention. Leads may be provided from the removal during development of poly-innervation where axonal expression of neuregulin1-III appears to be a sufficient signal for removal of the axons (Lee et al., 2016). At later stages, the re-innervation process becomes insufficient possibly owing to limitation to the enlargement of an MN’s terminal field evident also in young adult subjects (Rochel and Robbins, 1988).

Contrary to the consistency between studies on structural and molecular changes engaging the components of the NMJ, recordings of the transmission at the NMJ show contradictory results, with substantial variation within and between muscles and between species (reviewed in Willadt et al., 2018). Overall, there is not coherent evidence for transmission aberrations at the NMJ during aging. To some extent this may be due to unavoidable bias in the sampling of the NMJ to record from and thus that NMJs intact enough to lend themselves to electrophysiological examination are not seriously altered even at advanced age.

From the above it may be concluded that a large body of evidence indicates that disrupted innervation and concurrent re-innervation are present at an early preclinical phase of sarcopenia and that this re-organization due to drop out of axonal branches primarily impacts the integrity of the large fast twitch MUs. These changes instigated by the removal of dysfunctional endings take place in parallel with aging-caused aberrations of the peripheral axons and their myelin sheaths. Axon dystrophy and atrophy represents the most prominent signs of normal neuronal aging in both the central and peripheral nervous system (see above). It is therefore of considerable interest that genetic ablation of the FOXO transcription factors in mice accelerated aging-induced axonal degeneration and was accompanied by increased mTOR activity while this acceleration could be impeded by rapamycin (mTOR inhibitor) (Hwang et al., 2018). Recent work suggests that sirtuins, mTOR, and FOXOs may have significant roles in neurodegenerative diseases as well (Maiese, 2021). It is clear that we still have considerable knowledge gaps of the denervation process of myofibers and the underlying aging-induced degeneration of the motor axons. Furthermore, mapping of age of onset and pace of progression of sarcopenia to genetic polymorphisms (as FOXO3a, see above) is also missing. While awaiting progress in this research, it is encouraging that exercise, i.e., frequent use of the MUs, seems to slow down the aging of the connectivity between MNs and myofibers (Love et al., 2003; Soendenbroe et al., 2020). One contributing factor may be the higher levels of neurotrophic factors expressed in exercised over sedentary aged skeletal muscles (Mrówczyński, 2019; Stanga et al., 2020).

## The Sarcopenic Skeletal Muscle

Changes that take place in the skeletal muscle during aging have received comparatively more attention than muscle innervation, especially in studies of humans. The process that instigates sarcopenia is still the subject of debate (Gutman and Hanzlikova, 1972), at more advanced stages a multitude of alterations are present in peripheral innervation as well as in the target tissue, which makes it quite challenging to determine cause and effect. Most of our understanding of molecular and cellular mechanisms in myofibers and the muscle scaffold during aging stem from work done on laboratory animals. For a detailed account on myogenic mechanisms in the sarcopenia processes, we refer to some recent reviews (Larsson et al., 2019; Christian and Benian, 2020; Pascual-Fernández et al., 2020). Briefly, early studies on sarcopenia (previously referred to as old-age-muscle-atrophy or senile-muscle-atrophy) in laboratory small rodents and humans revealed that aging is accompanied by a loss of myofibers, atrophy preferentially affecting fast twitch (type II) myofibers, and a fiber type grouping—features that increase with advancing chronological age. Further, myofibers in aged subjects display irregularities of the end plate, increased content of lipid droplets and of secondary lysosomes, and an increasing number of fibers that show a myosin expression pattern intermediate between type I and type II (hybrid fibers) (Lexell et al., 1983; Bowen et al., 2015; Larsson et al., 2019). Concurrent alterations in the amount of fat and connective tissue, vasculature, and thickening of the capillary basement membrane have been described indicating that the muscle scaffold is impacted by the aging process (McGregor et al., 2014; Larsson et al., 2019). On most of these variables, there is a good agreement between observations made in humans and small rodents. For obvious reasons, a major obstacle in human studies is the limitation of performing longitudinal studies and hence most studies use a cross-sectional design. This design does not take into account the individual variation in factors that are known to have a huge impact on sarcopenia, such as genetic make-up, socio-economic changes, physical activity, and medical history (Mitchell et al., 2012).

## Changes to the Remodeling Capacity of Skeletal Muscles During Aging

In addition to the well-established age-related structural changes, another hallmark of the aged human muscle is a diminished muscle remodeling capacity. This will impact the possibility of the skeletal muscle to respond to age-associated changes in the innervation. An attenuated remodeling capacity is evidenced by the dissipating capacity to recover from immobilization-induced muscle mass loss with increasing age (Figure 3; Wall et al., 2013) and is further demonstrated by a diminished adaptation to both resistance and aerobic exercise with age. For example, it was recently reported that 12 weeks of heavy resistance training in very old men and women (age 83–94) had no effects on type II fiber area, satellite cell content, or myonuclear density, supporting the observation of reduced plasticity at advanced ages (Karlsen et al., 2019).

An important factor in this context is anabolic resistance, which refers to blunted protein synthesis in response to anabolic stimuli such as exercise and nutrients. Anabolic resistance has been attributable to attenuated TORC1- dependent and TORC1- independent mechanisms (e.g., MAPK/ERK pathways) and includes effects on ribosomal biogenesis (Cuthbertson et al., 2005; Fry et al., 2011). Other mechanisms that may participate in the progressive attenuation of remodeling capacity with age is the accumulation of somatic mutations in satellite cells (SCs; myogenic stem cells) in human muscles (Franco et al., 2019). An accumulation rate of 13 somatic mutations per genome and year was reported to occur and these mutations were also detected in the adult myofiber (Franco et al., 2018). The latter observation suggests that, through SC fusion, mutations propagate into the mature multinucleated muscle fiber and impact skeletal muscle cell function. In addition, several groups have reported a reduced number of SC in aged muscle, especially around type II fibers, concurrent with a reduced proliferation and differentiation capacity of the SCs (Verdijk et al., 2014; Franco et al., 2019). A linear correlation between number of SCs and fiber size has been reported in aged individuals (Mackey et al., 2011; Franco et al., 2019), and a reduced number of SCs seems to be associated with diminished muscle remodeling capacity (Suetta et al., 2013). This is further underlined by evidence that depletion of SCs in animal models leads to an increase in the amount of fibrotic connective tissue and adipocyte infiltration, two hallmarks of the aged skeletal muscle (Franco et al., 2019). From a mechanistic point of view, a blunted activation of SCs in aged individuals is observed in parallel with an attenuation of Notch signaling, which is possible to reverse through an activation of this pathway (Carlson et al., 2009; Suetta et al., 2013). An increased expression of the cellular senescence marker p16 has also been detected in SCs in aged individuals compared to young individuals (Carlson et al., 2009; Suetta et al., 2013). Other well-established age-associated changes in the muscle scaffold include alterations of the cellular composition (infiltration of inflammatory cells, adipocytes, and fibroblasts), as well as increased amounts of connective tissue and intra-myocellular lipids. This affects the microenvironment and the crosstalk between cells (Latroche et al., 2017). Moreover, the intra-myocellular lipids and their derivatives have been shown to induce mitochondrial dysfunction and increase generation of reactive oxygen species as well as the development of the age-related chronic low-grade systemic inflammation (Kalinkovich and Livshits, 2017). Changes in the micro-milieu are plausible mechanisms in both the dissipation of muscle function and the decreased ability to regenerate in response to various stimuli (Hiona and Leeuwenburgh, 2008; Pillon et al., 2013). Moreover, as observed in SCs from older individuals, changes in the micro-milieu could also introduce chromatin alterations and DNA methylation changes, which may add to changes induced by somatic mutations (Bigot et al., 2015; Franco et al., 2019).

By using participants of the population-based Uppsala longitudinal study of adult men (ULSAM) cohort (Skoglund et al., 2020), changes in skeletal muscle expression in a set of candidate genes involved in muscle remodeling was analyzed longitudinally. At age 70, an activation of the pathways associated with UPS was observed but this response was significantly attenuated by age 88. The gene expression pattern at 70 years was defined as beneficial since the subjects maintained their muscle fiber histology and appendicular lean body mass until advanced age. This is consistent with observations made in small rodents, where it was proposed to be related to an initial increased remodeling due to denervation/re-innervation followed by incapacitation at advanced age resulting in an increased number of permanently denervated myofibers (Altun et al., 2012). A similar response has recently been demonstrated in human skeletal muscle (Piasecki et al., 2018).

## How Do We Counteract Sarcopenia and Extend the Healthspan?

The literature clearly shows that both genetic and environmental factors are closely intertwined and together will define the individual’s phenotype at different ages. Both the onset and the progression of sarcopenia are highly variable between subjects owing to genetic and environmental/life-style factors (Degens and Korhonen, 2012; Bann et al., 2015; Khanal et al., 2020; Figures 3A,B). Muscle strength and the amount of muscle mass in adult life are to a large extent due to genetic factors; heritability estimates of >50% are not uncommon (Zempo et al., 2017). A number of genes such as ACTN3, ACE, and VDR have been associated with skeletal muscle phenotypes (Pratt et al., 2019; Singh and Gasman, 2020). GWAS studies have linked genetic polymorphisms to both muscle growth/lean body mass and [handgrip] strength. However, linkage to establish their importance to skeletal muscle-trait variation has generally been identified as small, especially with advancing age (Carmelli and Reed, 2000). Notably, while some of the so-far identified genes associated with the above metrics have a function in neuromuscular transmission and neuronal plasticity, most of the strongly associated genes have a hitherto unknown function in skeletal muscle (*idem*). A problem with these linkage/association studies is that they address metrics such as static handgrip strength and lean body mass, while the association between genes/genetic polymorphism and the age-of-onset and pace-of-progression of sarcopenia remains to be addressed.

Since population-based studies clearly demonstrate that environmental factors such as nutrition together with neuroendocrine changes are associated with loss of both muscle function and mass as well as the tissue integrity of skeletal muscle during aging, many intervention regimens have been targeted to correct or override these changes (Larsson et al., 2019). Physical activity in adult life will, in contrast to a sedentary life-style, preserve muscle function better at old age in both humans and rodents (Trappe et al., 1996; Bann et al., 2015; Drey et al., 2016; Figure 3). However, sarcopenia also affects top athletes (Trappe et al., 1996; Gava et al., 2015; Drey et al., 2016; Piasecki et al., 2016; Ganse et al., 2020) and analysis of data on track and field records (both cross-sectional and/or longitudinal records) of master athletes discloses that there is steady decline in power of about 1% per year between 30–70 years of age. At more advanced age, most datasets show an accelerated drop (1–2% per year) (Trappe et al., 1996; Gava et al., 2015; Ganse et al., 2020) and the slope of the decrease is steeper for ballistic power demanding sports than running sports (Gava et al., 2015). This re-emphasizes that recruitment/contraction speed and coordination may be more sensitive to aging than strength and mass and, further, that once sarcopenia becomes clinically overt the process has probably been ongoing for decades but clinically compensated for by intrinsic mechanisms. The significance of physical activity is, however, underscored by the fact that the loss of muscle mass and function is accelerated during periods of immobilization (Wall et al., 2013). This is further aggravated by the diminished muscle remodeling capacity of aged individuals in the recovery following mobilization-induced muscle loss (Suetta et al., 2013). Even though the response to exercise is to some extent blunted at older age, it is still the best documented intervention strategy (Bao et al., 2020; Grgic et al., 2020). There are conflicting opinions concerning the benefits of strength vs. endurance training or if a combination of both tailored to the individual is optimal (Trappe et al., 1996; Mitchell et al., 2012; Bowen et al., 2015; Drey et al., 2016; Karlsen et al., 2019). Resistance-type training has the largest impact on skeletal muscle mass but aerobic exercise also attenuates aging-induced changes and it preferentially stimulates metabolic pathways such as oxidative capacity including mitochondrial density, structure, and function. Importantly, exercise increases insulin sensitivity by increasing sarcolemmal glucose transporter (Glut 4) levels, augmenting metabolic flexibility, and reducing the risk of development of insulin resistance (type 2 diabetes) (Biensø et al., 2015; Bowen et al., 2015; Cartee et al., 2016). Exercise also decreases fat infiltration, increases SC number, and appears to facilitate the maintenance of the NMJ integrity at advanced age (Valdez et al., 2010; Power et al., 2016; Franco et al., 2019). Furthermore, increased physical activity has positive effects on age-related diseases linked to secondary sarcopenia such as chronic heart failure and obstructive lung diseases (Coats, 1996; Bernard et al., 1998).

It could be argued that exercise, and especially resistance exercise, activates mTOR pathways and one of key changes identified by CR/DR models is a reduced mTOR activity which seems to attenuate the aging process. An interesting observation in humans by Phillips et al. (2013) that individuals with the greatest increase in lean mass gain following resistance training had a suppressed mTOR signaling over the training period. Moreover, the overall effects of exercise are largely coherent with the findings in the CR models, with increased PGC-1 expression and activation of AMPK (Egan and Zierath, 2013; Bowen et al., 2015). More knowledge is needed to understand how the activities of these signal pathways contribute to the aging process in skeletal muscle and the effects of counteracting exercise regimes. Importantly not all individuals respond to the same extent to exercise (i.e., high versus low responders) and meta-analyses provide evidence that very old individuals remain responsive to resistance-type training with increases in strength that occurs without any changes in muscle mass (Beckwée et al., 2019; Karlsen et al., 2019). The latter suggests that exercise-induced changes in advanced age may be more related to neuromuscular function and/or intrinsic contractility machinery than increase in muscle mass (Biolo et al., 2014). This may have implications for the design of physical activity programs for the elderly population.

## Summary and Future Perspective

Sarcopenia is a facultative trait of the aged human. The underlying process of this disorder which becomes clinically overt during the 5th–7th decades of life may start as early as during the 3rd decade as judged by decreases in muscle strength and power. We argue that sarcopenia has a neurogenic origin while in the clinically manifest disorder the innervating MNs, target myofibers, and muscle scaffold are probably components of a viscous cycle were it is challenging to decide on what is cause or consequence. We have pointed to gaps in knowledge, such as the crosstalk between motor axons, terminal Schwann cells, and myofibers in the denervation/re-innervation processes that lead to a loss of MU and muscle weakness during aging. To capture the early events of sarcopenia, we will need to modify the operational definition and introduce the concept of the pre-clinical phase that should include more functional metrics and validated biomarkers for susceptibility to develop severe sarcopenia.

It is clear that the rapidly changing composition of the human population will impact incidence and prevalence of diseases and aging-induced disorders and that closing the gap between healthspan and lifespan should be our next priority. To address this we must fill knowledge gaps in our understanding of the biology of aging including sarcopenia. Linkage studies of genetic polymorphisms associated with age-of-onset and pace-of-progression of sarcopenia are missing but warranted to shed light on the large difference in impact between individuals and why some respond to interventions while others do not.

We argue that preventive measures to intercept with biological aging are lagging behind efforts to treat manifest clinical outcomes of aging. To accomplish compliance with prescription of preventive measures, we need to identify reliable predictors to convince individuals to enroll in intervention programs that may be life-long.

## Author Contributions

Both authors listed have made a substantial, direct and intellectual contribution to the work, and approved it for publication.

## Conflict of Interest

The authors declare that the research was conducted in the absence of any commercial or financial relationships that could be construed as a potential conflict of interest.

## Footnotes

1 https://grg.org/

2 Assuming menarche at 12 years, a 50–75% mortality of children and need of parental protection until puberty, and that starvation and prolonged breast feeding limited the frequency of pregnancies to every other year; a life span of 35–39 years is still enough to accomplish a net growth of the human population.
