# Supplementary material for: Sarcopenia: What Is the Origin of This Aging-Induced Disorder?
Source: Front Genet. 2021 Jul 2;12:688526. doi: 10.3389/fgene.2021.688526 (PMC8285098; doi:10.3389/fgene.2021.688526)
Supplement: Supplementary file 1 [file Data_Sheet_1.pdf]

Supportive information

Sarcopenia: what is the origin of this disorder?

T. Gustafsson and B. Ulfhake

Fig. S1 A-B

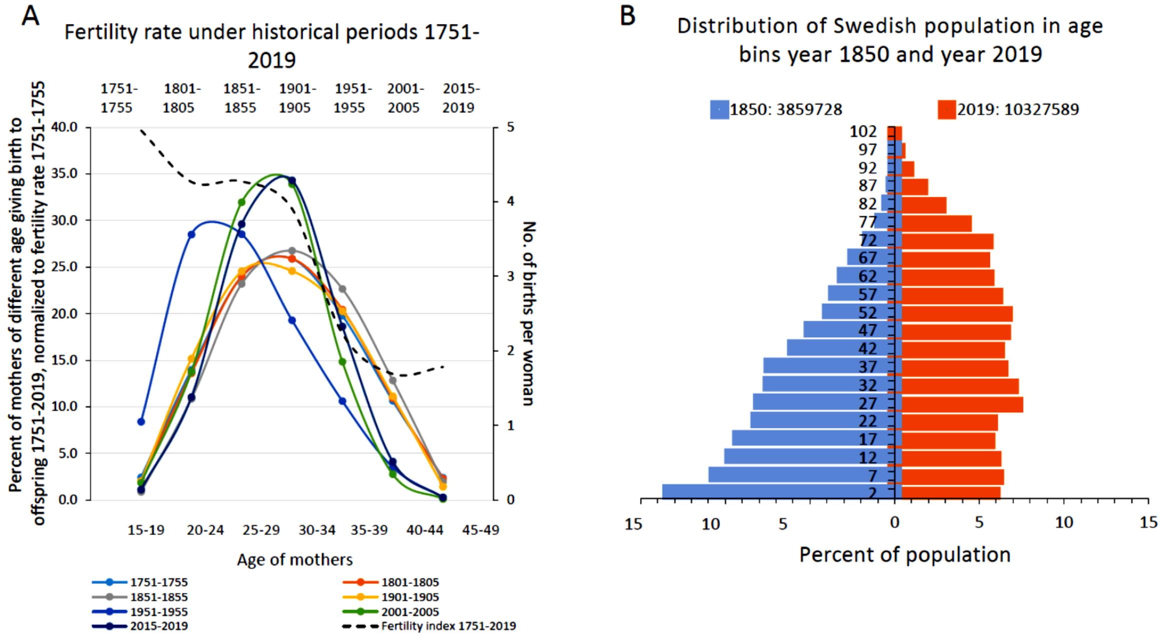

Fig. S1 A-B (A) shows the change in women's fertility rate from 1751 until 2019 (interrupted line; abscissa on the top and ordinate to the right) and frequency of child births (ordinate to the left) at different age of the mother (abscissa at the bottom) across the reproductive period divided in 5-year bins and starting at 1751 until 2019 (each period indicated by a different color; see key under diagram). Note that the fertility rate has been normalized to 1751 to facilitate comparison of reproductive behavior over time. Except for the mid-twentieth century during the dramatic drop in fertility rate, reproduction peak at ~30 years and is essentially unaltered between 1751 and 2019. Young and older mothers are, however, less common today, compressing the reproduction period. (B) shows the impact of life span extension 1850-2019 and the concomitant decrease in fertility rate on the distribution of the Swedish population in age bins 1850 and 2019, respectively. A-B are data replotted from[1, 2].

1. Population development in Sweden in a 250-year perspective, Statistics Sweden, 1999:2, ISBN 91-618-1021-5.
2. Population statistics, Statistical database, Sweden Statistics, available at www.http://www.statistikdatabasen.scb.se/pxweb/en/ssd/

Effects of calorie (dietary) restriction

Calorie/dietary restriction (CR/DR) is the most robust and general (across mammalian and non-mammalian species) intervention known to retard aging. As described by Speakman[1] *mens sana in corpore sana* is part of our cultural heritage to feed modestly and stay fit and lean, to provision for

health and growing old. However, it was not until laboratory work in the 20th century (for references see[1]) showing that reducing calorie or dietary (CR/DR restriction) intake by 10%-45% increased lifespan by up to 50% in many invertebrate and vertebrate species implicating that this intervention intercept with highly conserved mechanisms of systems biology involved in aging of organisms [2]. In mammals, CR reduces the incidence of common morbidities such as cardiovascular diseases and cancer (main causes of death at older ages in HICs) and, thus, seems to extend health span to a similar extent as lifespan. Another interesting feature is that this intervention, at least in some species, shows a dose-response relationship (both degree of restriction and time under restriction) with health- and lifespan extension (*idem*).

When initiated at young age (pre puberty) CR will retard growth compared with free feeding age matched controls. When administrated later (post adolescence or middle ages) mice and rats will lose some of the starting whole body weight while adapting to CR [3,4] which affect both lean body mass and, in particular, adipose tissue[5-8]. After this adaptation at least small rodents maintain a very stable body weight throughout most of the lifespan (*idem*). The impact of CR on the lean body mass at later ages is mainly on the musculoskeletal system because beyond 3-6 months of age most internal organs like lungs, liver and kidneys have ceased to growth and adaptive responses are small compared with muscle and fat tissues[9,10]. Small rodents are endothermic and respond to CR with a lowering of average daily body temperature and torpor[6-8,11,12]. These adaptations are accompanied by alterations in the endocrine system with decreased levels of TSH,T4 and T3[13-15] (HPT axis), growth hormone and IGF-1, and lower levels of blood glucose and insulin as well as increased insulin sensitivity [8,16-18]. Metabolically, there is a shift from glycolytic (glucose) towards lipolytic (fatty acids, β-oxidation) metabolism [19] and indirect calorimetry has shown that subjects under CR synthetize fatty acids during the postprandial period to be energy substrates during the period without access to food. In subjects who maintain CR, the increase in metabolic activity of the over-all reduced amount of white adipose tissue (WAT) is accompanied by an upregulation of key lipid enzymes[19,20] and a browning of the white fat[20]. While the size of the remaining adipose tissues may be important to reach optimal benefits from CR at least in-between strains of laboratory mice[21,22], the overall decrease will alter adipose tissue signalling with lower levels of leptin and increased levels of adiponectin in proportion to the reduction of WAT[23]. The metabolic shift has also been argued to be the main reason for the lower levels of oxidative (ROS) stress in subjects under CR[1,24,25] providing a partial rescue from molecular damage, including DNA, which is considered a main factor driving organismal aging[1,16,17]. Under CR the decreased production of ATP (higher AMP:ATP ratio) and increased adiponectin signalling will both induce AMP-activated-protein-kinase (AMPK)[23] which will enhance mitochondrial biogenesis, p53 activity and depress anabolic drive by inhibition of mTOR (*idem*). mTOR (mammalian/mechanistic target of rapamycin) is a key protein of the TORC complex (TORC1/2) serving as a platform regulating cell growth by promoting protein translation and inhibiting the FOXO family (1,3 and 4) of transcription factors (*idem*). Tyrosine kinase receptor (TKR) activation can induce TORC activity and an important modulator of mTOR which is depressed under CR is the signalling through IGF-1/insulin-insulin/IGF-TKR-insulin response element IRS1/2. In addition to inhibiting mTOR, CR induces also another family of proteins, the sirtuins (Sirt 1-6)[26,27]. The sirtuins (nicotinamide adenine dinucleotide (NAD)-dependent protein deacetylases) are metabolic sensors that stimulate mitochondrial biogenesis through PGC-1α, lower oxidative stress, inhibit mTOR and induce both FOXO and autophagy-lysosomal activity, reduce oxidative stress and DNA damage and increase p53 activity[28-32]. At least Sirt 1 and 3 seem to be non-redundant for the response to CR in

mice[28,29,31]. Both sirtuins and the inhibition of mTOR will activate the FOXO family of transcription factors paving the way for increased regulated proteolysis through the ubiquitin-proteasome system (UPS) and increased degradation of proteins, molecular complexes and organelles through the autophagy-lysosomal pathway conferring a more rapid turn-over of cellular constituents[33-35]. FOXO also upregulates cellular oxidative defence and have an inhibiting function on proteins involved in cell cycling (*idem*). Combined, the CR mediated decrease in oxidative stress, increased biogenesis of mitochondria and accelerated turn-over of cellular constituents may slow the wear and tear that generates the cumulative build-up of unrepaired damage during aging. The possibility that the beneficiary effects of CR may be co-mediated by the microbiome has not yet been sufficiently explored but existing data indicates that aging brings about changes to the composition of the microbiota leading to a dysbiosis that may contribute significantly to the aging process[36] (Fig. 2). . The extent of energy saving contributed by a decreased physical activity is still being the subject of debate[4-8,11,37]. However, during periods of rest CR subjects have a lower body temperature than controls and resting CR mice often enter turpor, implicating that energy is saved by a lower resting metabolic rate (RMR). Trials with chemical compounds that show promising extension of life span in model organisms are all partial mimics of CR and targets mTOR (rapamycin[38,39]), AMPK (metformin[40]) or indirectly both sirtuins and AMPK by inhibiting mitochondrial function (resveratol[41]).
Trials with CR on long-lived species as primates[42-45] and humans[46,]remain for obvious reasons limited but show so far promising outcomes and suggest that CR mediates its effects through the same evolutionary conserved mechanisms as described above for small rodents and that several of the gene variants associated with human longevity[17] are genes implicated in the beneficial outcome of CR, such as FOXO(3A),the growth hormone-IGF-1 signal transduction pathway and proteins involved in cell cycling [47,48].

References

1. Speakman JR, Mitchell SE. Caloric restriction. *Mol Aspects Med.* 2011;32(3):159-221.
2. Fischer AW, Cannon B, Nedergaard J. Optimal housing temperatures for mice to mimic the thermal environment of humans: An experimental study. *Molecular metabolism.* 2018;7:161-170.
3. Altun M, Bergman E, Edstrom E, Johnson H, Ulfhake B. Behavioral impairments of the aging rat. *Physiol Behav.* 2007;92(5):911-923.
4. Fahlstrom A, Zeberg H, Ulfhake B. Changes in behaviors of male C57BL/6J mice across adult life span and effects of dietary restriction. *Age (Dordr).* 2012;34(6):1435-1452.
5. Duffy PH, Feuers RJ, Hart RW. Effect of chronic caloric restriction on the circadian regulation of physiological and behavioral variables in old male B6C3F1 mice. *Chronobiology international.* 1990;7(4):291-303.
6. Duffy PH, Leakey JE, Pipkin JL, Turturro A, Hart RW. The physiologic, neurologic, and behavioral effects of caloric restriction related to aging, disease, and environmental factors. *Environmental research.* 1997;73(1-2):242-248.
7. Gluck EF, Stephens N, Swoap SJ. Peripheral ghrelin deepens torpor bouts in mice through the arcuate nucleus neuropeptide Y signaling pathway. *Am J Physiol Regul Integr Comp Physiol.* 2006;291(5):R1303-1309.
8. Cameron KM, Golightly A, Miwa S, Speakman J, Boys R, von Zglinicki T. Gross energy metabolism in mice under late onset, short term caloric restriction. *Mech Ageing Dev.* 2011;132(4):202-209.

9. Lessard-Beaudoin M, Laroche M, Demers MJ, Grenier G, Graham RK. Characterization of age-associated changes in peripheral organ and brain region weights in C57BL/6 mice. *Exp Gerontol.* 2015;63:27-34.
10. Lui JC, Forcinito P, Chang M, Chen W, Barnes KM, Baron J. Coordinated postnatal down-regulation of multiple growth-promoting genes: evidence for a genetic program limiting organ growth. *FASEB J.* 2010;24(8):3083-3092.
11. Hambly C, Speakman JR. Contribution of different mechanisms to compensation for energy restriction in the mouse. *Obes Res.* 2005;13(9):1548-1557.
12. Rikke BA, Johnson TE. Physiological genetics of dietary restriction: uncoupling the body temperature and body weight responses. *Am J Physiol Regul Integr Comp Physiol.* 2007;293(4):R1522-1527.
13. Araujo RL, Andrade BM, da Silva ML, Ferreira AC, Carvalho DP. Tissue-specific deiodinase regulation during food restriction and low replacement dose of leptin in rats. *Am J Physiol Endocrinol Metab.* 2009;296(5):E1157-1163.
14. Araujo RL, de Andrade BM, de Figueiredo AS, et al. Low replacement doses of thyroxine during food restriction restores type 1 deiodinase activity in rats and promotes body protein loss. *The Journal of endocrinology.* 2008;198(1):119-125.
15. Lamberts SW, van den Beld AW, van der Lely AJ. The endocrinology of aging. *Science.* 1997;278(5337):419-424.
16. López-Otín C, Blasco MA, Partridge L, Serrano M, Kroemer G. The hallmarks of aging. *Cell.* 2013;153(6):1194-1217.
17. Partridge L, Deelen J, Slagboom PE. Facing up to the global challenges of ageing. *Nature.* 2018;561(7721):45-56.
18. Azzu V, Valencak TG. Energy Metabolism and Ageing in the Mouse: A Mini-Review. *Gerontology.* 2017;63(4):327-336.
19. Bruss MD, Khambatta CF, Ruby MA, Aggarwal I, Hellerstein MK. Calorie restriction increases fatty acid synthesis and whole body fat oxidation rates. *Am J Physiol Endocrinol Metab.* 2010;298(1):E108-116.
20. Fabbiano S, Suarez-Zamorano N, Rigo D, et al. Caloric Restriction Leads to Browning of White Adipose Tissue through Type 2 Immune Signaling. *Cell Metab.* 2016;24(3):434-446.
21. Rikke BA, Yerg JE, 3rd, Battaglia ME, Nagy TR, Allison DB, Johnson TE. Strain variation in the response of body temperature to dietary restriction. *Mech Ageing Dev.* 2003;124(5):663-678.
22. Liao CY, Rikke BA, Johnson TE, Gelfond JA, Diaz V, Nelson JF. Fat maintenance is a predictor of the murine lifespan response to dietary restriction. *Aging Cell.* 2011;10(4):629-639.
23. Hardie DG. Sensing of energy and nutrients by AMP-activated protein kinase. *Am J Clin Nutr.* 2011;93(4):891S-896.
24. Drew B, Phaneuf S, Dirks A, et al. Effects of aging and caloric restriction on mitochondrial energy production in gastrocnemius muscle and heart. *Am J Physiol Regul Integr Comp Physiol.* 2003;284(2):R474-480.
25. Bevilacqua L, Ramsey JJ, Hagopian K, Weindruch R, Harper ME. Long-term caloric restriction increases UCP3 content but decreases proton leak and reactive oxygen species production in rat skeletal muscle mitochondria. *Am J Physiol Endocrinol Metab.* 2005;289(3):E429-438.
26. Guarente L. Calorie restriction and SIR2 genes--towards a mechanism. *Mech Ageing Dev.* 2005;126(9):923-928.
27. Guarente L. Sirtuins, aging, and metabolism. *Cold Spring Harb Symp Quant Biol.* 2011;76:81-90.
28. Bordone L, Cohen D, Robinson A, et al. SIRT1 transgenic mice show phenotypes resembling calorie restriction. *Aging Cell.* 2007;6(6):759-767.
29. Boily G, Seifert EL, Bevilacqua L, et al. SirT1 regulates energy metabolism and response to caloric restriction in mice. *PLoS One.* 2008;3(3):e1759.

30. Chen D, Bruno J, Easlon E, et al. Tissue-specific regulation of SIRT1 by calorie restriction. *Genes Dev.* 2008;22(13):1753-1757.
31. Someya S, Yu W, Hallows WC, et al. Sirt3 mediates reduction of oxidative damage and prevention of age-related hearing loss under caloric restriction. *Cell.* 2010;143(5):802-812.
32. Salminen A, Kaarniranta K. SIRT1: regulation of longevity via autophagy. *Cell Signal.* 2009;21(9):1356-1360.
33. Martins R, Lithgow GJ, Link W. Long live FOXO: unraveling the role of FOXO proteins in aging and longevity. *Aging cell.* 2016;15(2):196-207.
34. Calissi G, Lam EW, Link W. Therapeutic strategies targeting FOXO transcription factors. *Nat Rev Drug Discov.* 2021;20(1):21-38.
35. Maiese K. Targeting the core of neurodegeneration: FoxO, mTOR, and SIRT1. *Neural regeneration research.* 2021;16(3):448-455.
36. Kundu P, Lee HU, Garcia-Perez I, et al. Neurogenesis and prolongevity signaling in young germ-free mice transplanted with the gut microbiota of old mice. *Science translational medicine.* 2019;11(518).
37. Duffy PH, Feuers RJ, Leakey JA, Nakamura K, Turturro A, Hart RW. Effect of chronic caloric restriction on physiological variables related to energy metabolism in the male Fischer 344 rat. *Mech Ageing Dev.* 1989;48(2):117-133.
38. Harrison DE, Strong R, Sharp ZD, et al. Rapamycin fed late in life extends lifespan in genetically heterogeneous mice. *Nature.* 2009;460(7253):392-395.
39. Miller RA, Harrison DE, Astle CM, et al. Rapamycin-mediated lifespan increase in mice is dose and sex dependent and metabolically distinct from dietary restriction. *Aging Cell.* 2014;13(3):468-477.
40. Weiss R, Fernandez E, Liu Y, Strong R, Salmon AB. Metformin reduces glucose intolerance caused by rapamycin treatment in genetically heterogeneous female mice. *Aging (Albany NY).* 2018;10(3):386-401.
41. Price NL, Gomes AP, Ling AJ, et al. SIRT1 is required for AMPK activation and the beneficial effects of resveratrol on mitochondrial function. *Cell Metab.* 2012;15(5):675-690.
42. Colman RJ, Anderson RM, Johnson SC, et al. Caloric restriction delays disease onset and mortality in rhesus monkeys. *Science.* 2009;325(5937):201-204.
43. Fowler CG, Chiasson KB, Leslie TH, et al. Auditory function in rhesus monkeys: effects of aging and caloric restriction in the Wisconsin monkeys five years later. *Hear Res.* 2010;261(1-2):75-81.
44. Kastman EK, Willette AA, Coe CL, et al. A calorie-restricted diet decreases brain iron accumulation and preserves motor performance in old rhesus monkeys. *J Neurosci.* 2010;30(23):7940-7947.
45. McKiernan SH, Colman RJ, Lopez M, et al. Caloric restriction delays aging-induced cellular phenotypes in rhesus monkey skeletal muscle. *Exp Gerontol.* 2011;46(1):23-29.
46. Fontana L, Klein S, Holloszy JO, Premachandra BN. Effect of long-term calorie restriction with adequate protein and micronutrients on thyroid hormones. *J Clin Endocrinol Metab.* 2006;91(8):3232-3235.
47. Guevara-Aguirre J, Guevara A, Palacios I, Pérez M, Prócel P, Terán E. GH and GHR signaling in human disease. *Growth Horm IGF Res.* 2018;38:34-38.
48. Guevara-Aguirre J, Rosenbloom AL. Obesity, diabetes and cancer: insight into the relationship from a cohort with growth hormone receptor deficiency. *Diabetologia.* 2015;58(1):37-42.
